# WMambaFuse: an infrared and visible image fusion network based on wavelet mamba

**DOI:** 10.1038/s41598-026-44374-y

**Published:** 2026-03-18

**Authors:** Jiashuo Wang, Yu Si, Yong Chen, Xiaoyun Sun, Zhiyu Li, Hui Xing, Mingming Wang

**Affiliations:** 1 School of Mechanical Engineering, Shijiazhuang Tiedao University, Shijiazhuang, 050043 China; 2 CRSC Low-Altitude Intelligence Technology Co., Ltd., Beijing, 100000 China; 3 North China Institute of Aerospace Engineering, Langfang, 065000 China; 4 School of Electrical and Electronic Engineering, Shijiazhuang Tiedao University, Shijiazhuang, 050043 China; 5 Hebei Provincial Collaborative Innovation Center of Transportation Power Grid Intelligent Integration Technology and Equipment, Shijiazhuang Tiedao University, Shijiazhuang, 050043 China

**Keywords:** Infrared image, Visible image, Image fusion, Wavelet transform, Mamba, Spatial-frequency fusion, Engineering, Mathematics and computing

## Abstract

Infrared and visible image fusion aims to generate a fusion image that integrates complementary information from both modalities. Current deep learning-based methods often exhibit a limitation: networks that focus on spatial-domain features usually neglect the detail-enhancing potential of frequency-domain information, whereas purely frequency-domain approaches struggle to maintain semantic coherence with spatial structures. To address this, we propose a novel and efficient fusion model. First, a Swin Transformer-based encoder is employed to extract deep, multi-scale features. Second, a complementary dual-branch “Spatial-Frequency” fusion module is designed. This module comprises a Spatial Attention Fusion Module (SAFM) for refining features in the spatial domain and a novel Wavelet-domain Mamba Fusion Module (WMFM) for processing features in the frequency domain, where the Mamba mechanism synergistically integrates global semantics and local details. Finally, a recurrent neural network architecture is employed in the decoder to preserve the coherence of spatio-temporal information. Quantitative experimental results on three public datasets (TNO, RoadScene, and MSRS) demonstrate that the proposed method achieves top or near-top rankings across multiple evaluation metrics, including ${FMI}_{wt}$, AG, VIF, SSIM, and SCD. These results clearly demonstrate that the proposed fusion method effectively preserves high-frequency details and edge information from source images, yielding fused images with superior visual quality and structural fidelity. This further validates the effectiveness and robustness of the proposed fusion strategy.

## Introduction

Visible-light images exhibit high spatial resolution and rich color information, yet they are susceptible to degradation under low illumination or adverse weather conditions. In contrast, infrared images offer all-weather operational capability. However, they generally suffer from lower spatial resolution and present textural details with relative ambiguity. Infrared and Visible Image Fusion (IVIF) technology leverages the complementary characteristics of the two modalities. It effectively enhances infrared thermal signatures in the scene while preserving the detailed textural information inherent to visible-light images. Currently, IVIF technology is widely applied across numerous fields, including military reconnaissance^1^, remote sensing monitoring^2^, medical image analysis^3^, and target tracking^4^.

IVIF methods can generally be categorized into traditional image fusion methods and deep learning-based fusion methods. Among traditional approaches, techniques include subspace-based methods^5,6^, saliency analysis methods^7–9^, and frequency-domain-based fusion methods^10–17^. Although conventional methods are computationally efficient, their performance in complex scenes is often limited due to reliance on manually designed fusion rules. In recent years, deep learning-based image fusion techniques have made significant progress. They can be primarily classified into four categories: methods based on autoencoders (AEs), convolutional neural networks (CNNs), generative adversarial networks (GANs), and Transformer-based approaches.

CNN-based methods use convolutional neural networks to extract image features, followed by reconstruction via deconvolution after fusion. The main advantage of this method lies in its powerful feature-learning capabilities, which can overcome the limitations of handcrafted rules and achieve superior fusion performance in complex scenarios. Prominent models in this category include U2Fusion^18^, DATFuse^19^, and SCGAFusion^20^. However, its drawbacks include a strong reliance on high-quality training data, limited interpretability, and the potential to introduce artifacts.

GAN-based methods consist of a generator and a discriminator, with the discriminator optimizing the quality of the fused image through an adversarial game with the generator. Prominent models in this category include FusionGAN^21^, DDcGAN^22^, AttentionFGAN^23^, and DUGAN^24^. However, the training instability of this method may lead to artifacts and loss of detail in the generated images.

Benefiting from its powerful global context modeling capability, the Transformer architecture has gained widespread attention in infrared and visible image fusion (IVIF) in recent years. Ma et al.^25^ proposed SwinFusion, which integrates CNN and Swin Transformer for efficient, flexible fusion. Zhao et al.^26^ designed CDDFuse, a dual-branch network. It uses a Base Transformer Encoder (BTE) and a Detail CNN Encoder (DCE) to separately extract low-frequency and high-frequency features, thereby mitigating detail loss. Wang et al.^27^ presented CrossFuse, which employs cross-attention to enhance the fusion of complementary information. However, this approach suffers from information loss and high computational cost. Yan et al.^28^ proposed ATFusion. Its feature fusion module independently extracts differential and standard features and uses cross-attention to balance texture and brightness. While achieving outstanding results, this model nonetheless tends to lose edge details.

Recently, Structured State Space Models (SSMs) have emerged as an efficient modeling approach in computer vision, gaining significant attention for their linear computational complexity and their ability to capture long-range dependencies. As a breakthrough, Mamba^29^ introduces a selective mechanism that not only overcomes the limitations of CNNs in capturing global contextual information but also avoids the computational complexity associated with Transformer self-attention. Mamba has been widely applied to general visual modeling^30–32^, image restoration^33^, and image fusion^34^, demonstrating its excellent scalability and potential for tackling complex visual tasks.

Despite significant progress, existing image fusion methods still face critical limitations. (1) Most fusion strategies rely primarily on weight allocation or convolutional operations. This approach often fails to effectively leverage contextual correlations among multimodal images. (2) A fundamental trade-off exists between global context modeling and local detail preservation. CNN-based methods effectively capture local spatial details but struggle with long-range dependencies, resulting in inconsistencies in global structures (e.g., thermal object contours). In contrast, Transformers excel at capturing global context via self-attention, yet they incur high computational costs and show limited sensitivity to high-frequency textures. (3) Most methods are confined to the spatial domain, neglecting the frequency domain’s advantages in parsing structures, edges, and textures. Although purely frequency-domain approaches have been explored, they typically sever the semantic link between spatial structures and frequency components. This failure to establish an effective cross-domain mapping ultimately limits further gains in fusion performance.

To address these challenges, we propose a novel fusion framework that synergistically leverages spatial and frequency-domain information. Specifically, it consists of two complementary branches: a spatial branch that extracts and fuses locally salient features, and an innovative frequency-domain branch designed with “directional decoupling—directional enhancement” capabilities. By integrating the wavelet transform with a multi-directional Mamba scanning mechanism, this branch achieves targeted enhancement and fusion of image edge and texture information. Through their collaboration, the framework achieves a more balanced and robust fusion of infrared thermal targets and visible-light texture backgrounds.

The main contributions of this paper are summarized as follows:

1. We propose A novel fusion method for visible and infrared images, which includes a Swin Transformer encoder, a recurrent decoder, and a dual-branch “spatial-frequency” fusion module. The multi-head self-attention mechanism of the Swin Transformer is used to extract multi-scale features across different modalities. The recurrent decoder is employed to reconstruct the fused image in both spatial and temporal dimensions.
2. A novel dual-branch complementary fusion network architecture is proposed, which consists of a Spatial Attention Fusion Module (SAFM) and a Wavelet-Mamba Fusion Module (WMFM) working collaboratively. The SAFM focuses on modeling local spatial details, while the WMFM captures edge and texture features in the frequency domain. These two branches are adaptively integrated via learnable weights, achieving complementary advantages in local details and global structural information, as well as robust feature fusion.
3. An innovative Wavelet-Mamba Fusion Module (WMFM) is designed. By introducing the wavelet transform for directional frequency-domain decomposition of features and integrating a multi-directional scanning state-space model (Mamba) to capture long-range dependencies across frequency bands, the module significantly improves the expressive power of edge and texture features.
4. Ablation and comparative experiments on public datasets confirm that our method surpasses existing state-of-the-art methods, achieving superior detail preservation, higher contrast, and better overall fidelity in both subjective and objective evaluations.

The remainder of this paper is structured as follows: Section “Related work” provides a review of fusion-related research; Section “Methods” elaborates on the proposed fusion framework in detail; Section “Experiments and result analysis” presents the experimental results; and Section “Conclusions” concludes the paper.

## Related work

This section primarily introduces image fusion methods based on the AE, Mamba, and wavelet transforms.

### Image fusion based on autoencoder (AE)

AE-based methods use a pre-trained encoder to extract features from the source image, and a decoder to reconstruct the fused image. Based on architectural features and fusion strategies, AE-based IVIF methods can be primarily divided into two generations:

1. *Hand-Crafted Rule-Based Fusion AE Methods* These methods focused on optimizing efficient encoder-decoder architectures, with feature fusion primarily relying on handcrafted strategies (such as average-pooling, max-pooling, etc.). For example, DenseFuse^35^ introduced dense blocks (Dense Blocks) to enhance detail preservation through feature reuse. NestFuse^36^ employs nested connections to construct a multi-scale feature pyramid, thereby enhancing feature fusion across different levels. Res2Net^37^(2021) replaced traditional dense blocks with multi-scale convolutional units and incorporated attention mechanisms to capture cross-modal complementary information. Res2Fusion^38^ integrates Res2Net and dense connections into the encoder to extract multi-scale features while preserving as much information as possible for the fusion task. Although these early methods established the foundation for AE-based IVIF, their reliance on handcrafted fusion rules limited their adaptability in complex scenarios.
2. *Learnable Fusion AE Methods* The key idea of this generation is to replace the fixed, handcrafted fusion rules in traditional AEs with trainable fusion modules. This shift has established learnable fusion as a mainstream paradigm in image fusion. DIVFusion (2023)^39^ addressed nighttime IVIF tasks by proposing a Scene-Illumination Disentangled Network (SIDNet) to mitigate illumination degradation in nighttime visible images. It also employed a Texture–Contrast Enhancement Fusion Network (TCEFNet) with attention-guided fusion strategies to integrate contrast and texture features, thereby generating colorful, detail-rich fusion results. In recent years, research has shifted toward developing trainable fusion modules and integrating advanced paradigms, such as Masked Autoencoders (MAE), contrastive learning, and Mamba, for adaptive cross-modal feature fusion. MaeFuse (2024)^40^ used a pre-trained Masked Autoencoder (MAE) as the encoder to simultaneously extract high-level semantic features and low-level texture information. Its fusion network comprised a Comparative Fusion Module (CFM) and a Merging Fusion Module (MFM), leveraging cross-attention and contrastive learning for interactive feature fusion. Zhang et al.^41^ proposed a novel gradient-based autoencoder fusion network (GuideFuse). This network is designed with a dedicated GuideValue (GV) to guide the decoder in reconstructing images. Meanwhile, a new fusion strategy based on computed GV is proposed, achieving excellent fusion performance.

While learnable fusion methods have made significant progress, they still face core challenges, including limited model generalization, high computational complexity, insufficient use of frequency-domain information, and difficulty balancing features across modalities.

### Image fusion based on mamba

The core of Mamba is the state space model (SSM)^42,43^, whose fundamental mechanism maps a one-dimensional input signal $x(t)\in {\mathbb{R}}^{N}$ to a one-dimensional output signal $y(t)\in {\mathbb{R}}^{N}$ via a hidden state vector $h(t)\in {\mathbb{R}}^{N}$, where *N* denotes the state dimension. This model can be expressed by the following linear ordinary differential equations:

1 $$\left\{ {\begin{array}{*{20}c} {h^{\prime}\left( t \right) = Ah\left( t \right) + Bx\left( t \right)} \\ {y\left( t \right) = Ch\left( t \right) + Dx\left( t \right)} \\ \end{array} } \right.$$

where $h(t)$ represents the hidden state vector at time *t*, $x(t)$ denotes the one-dimensional input signal, and $y\left(t\right)$ denotes the one-dimensional output signal. $A\in {\mathbb{R}}^{N\times N}$ is the state transition matrix, and $B\in {\mathbb{R}}^{N\times 1}$, $C\in {\mathbb{R}}^{1\times N}$,$D\in {\mathbb{R}}^{1}$ are the projection parameters.

To efficiently implement the SSM in deep learning, Mamba^29^ discretizes the aforementioned continuous system. The continuous parameters $A$ and $B$ are discretized using the zero-order hold (ZOH) method, as shown in Eqs. (2) and (3).

2 $$\overline{A} = \exp \left( {{\Delta }A} \right)$$

3 $$\overline{B} = ({\Delta }A)^{ - 1} \left( {{\mathrm{exp}}\left( {{\Delta }A} \right) - I} \right) \cdot {\Delta }B$$

where Δ is the timescale parameter, $\mathrm{exp}\left(\cdot \right)$ denotes the matrix exponential function, and $I$ represents the identity matrix. The discretized system can be expressed in the following form:

4 $$h_{k} = \overline{A}h_{k - 1} + \overline{B}x_{k}$$

5 $$\begin{array}{*{20}c} {} & {y_{k} = Ch_{k} + Dx_{k} } & {} & {} \\ \end{array}$$

where ${h}_{k}$ represents the state at the *k* discrete time step,${x}_{k}$ is the input at the *k* discrete time step, and ${y}_{k}$ is the output at the *k* discrete time step.

Equations 4 and 5 can be transformed into convolutional form (Eqs. 6 and 7).

6 $$\overline{K} = \left( {C\overline{B},C\overline{A}\overline{B}, \ldots ,C\overline{A}^{L - 1} \overline{B}} \right)$$

7 $$y = x{ \circledast }\overline{K}$$

where $L$ is the length of the input sequence, ⊛ denotes the convolution operation, and $\overline{K} \in {\mathbb{R}}^{L}$ is a structured convolutional kernel, $x$ denotes the input sequence.

Building on Mamba, Xie et al.^34^ introduced a dynamically feature-enhanced multimodal fusion method for Mamba images (FusionMamba). This method improves the performance of the visual state-space model Mamba by leveraging dynamic convolution and channel attention. It also strengthens texture features through the Dynamic Feature Fusion Module (DFFM). In various multimodal image fusion tasks and downstream experiments, the method demonstrates excellent applicability and superiority. Zhang et al.^44^ proposed a wavelet-based Mamba image enhancement model (CWNet). This network employs the wavelet transform as its backbone and incorporates a Mamba module to enhance high-frequency features, thereby achieving more accurate and robust image enhancement. Dong et al.^45^ proposed an improved multimodal fusion method. This method introduces a Fusion Mamba Module (FMB) that maps multimodal features into a hidden state space for interaction, aiming to reduce modal differences and enhance fused features. Zhou et al.^46^ proposed MaDiNet, which is the first to integrate a diffusion model with the Mamba state-space model for SAR target detection. They also designed the MambaSAR module to suppress interference from complex backgrounds. This method validates the effectiveness and generalizability of the diffusion-state-space framework for SAR detection. Li et al.^47^ proposed a dual-stage fusion model based on Mamba, named MambaDFuse. In this network, low-level features are extracted using a CNN, and high-level features are extracted using Mamba. An enhanced multimodal Mamba (M3) module is employed for deep-level fusion, effectively integrating complementary information across modalities. Finally, the fusion result is obtained by inverting the transformation applied to the extracted features. This method has achieved favorable results in both infrared–visible image fusion and medical image fusion tasks.

### Image fusion based on wavelet transform

The Wavelet Transform (WT) is a fundamental tool for time–frequency analysis. It decomposes an image into multiple subbands at different scales (frequencies) and orientations, such as horizontal, vertical, and diagonal, by convolving the image with a set of wavelet basis functions. The low-frequency subbands capture the overall structure and contours of the image, while the high-frequency subbands retain fine details such as edges and textures. This multi-resolution analysis, which simultaneously localizes information in both frequency and space, has made WT a widely adopted technique for image processing.

Xu et al.^48^ first introduced the Haar wavelet transform into a feature-extraction network as a replacement for traditional down-sampling, thereby significantly mitigating information loss. In 2024, Yang et al.^49^ proposed SFFNet, which employs the Haar wavelet to simultaneously capture local details and global structure, achieving significant performance gains in remote sensing image classification. In the same year, Wang et al.^50^ proposed FreqGAN, which substantially improved the fusion quality of infrared and visible images via a frequency-domain adversarial learning strategy. In 2025, the Fender team^51^ further developed a novel backbone network based on the Haar wavelet transform. As a result, state-of-the-art performance is achieved on multimodal image fusion benchmarks, and the effectiveness and broad prospects of frequency-domain feature fusion are fully validated. Sang et al.^52^ proposed CWFusion, an image fusion network for pedestrian detection that combines infrared and visible images. In this method, the input images are first decomposed into low-frequency and high-frequency components via the discrete wavelet transform. The detailed representations of the high-frequency components are then enhanced using a cross-attention mechanism. The network ultimately reconstructs images by fusing these components, thereby enabling effective infrared–visible image fusion for nighttime pedestrian detection. In 2025, Zhu et al.^53^ proposed a cross-modality fusion and object detection method, WaveMamba, to address the feature complementarity challenge in visible and infrared image object detection. This method employs the Discrete Wavelet Transform to decompose dual-modality images in the frequency domain and leverages the designed WaveMamba fusion module to collaboratively fuse low- and high-frequency sub-bands for object detection. Experimental results demonstrate that this method significantly improves the accuracy of dual-modality object detection. Chen et al.^54^ proposed the Mask-Guided Frequency Feature Fusion (MGFF) framework for RGB–IR object detection in remote sensing. This method utilizes the Wavelet Transform to decompose and enhance the frequency features of dual modalities, effectively reducing the modality discrepancy between visible and infrared images. Additionally, a multi-directional cross-modality perception fusion module is designed to achieve deep fusion of multi-modal information and to accomplish object detection. Experiments validate that this method achieves superior performance in both detection accuracy and robustness. This method also achieves state-of-the-art (SOTA) performance on both public datasets and the ATRNet-STAR dataset^55^.

Notably, diffusion models, leveraging their powerful generative priors, can not only preserve the overall structure of images but also generate rich high-frequency texture details, offering new research directions for image super-resolution and image fusion. Diao et al.^56^ proposed a dual-condition-guided diffusion network for remote sensing image registration and fusion (RFDifNet). This method comprises a registration diffusion module and a fusion diffusion module, which jointly optimize registration and fusion via a closed-loop iterative mechanism, demonstrating superior performance in geometric correction and detail preservation. Jie et al.^57^ introduced a semantic guidance and clarity-aware joint image fusion and super-resolution method (FS-Diff). By leveraging a clarity sensing mechanism to provide semantic guidance, this approach enables cross-modal feature extraction under low-resolution conditions. Simultaneously, using source images and their semantic information as conditions, an improved U-Net iteratively denoises across multiple noise levels, ultimately generating high-resolution fused images that integrate cross-modal features and rich semantic information. Yang et al.^58^ proposed a latent feature-guided diffusion Transformer model for general image fusion (LFDT-Fusion). The model consists of a U-Net-based spatial autoencoder and a Transformer-based diffusion network: the former extracts image features, while the latter captures long-range dependencies and complex cross-domain information among features, enabling high-quality feature interaction and fusion. This method establishes an efficient, stable, and high-performance framework for general image fusion.

Furthermore, recent advancements in other subfields of deep learning have also provided novel insights into cross-modal feature fusion. Zhu et al.^59^ proposed a global-detail dual-branch super-resolution network (GDSR) to address the challenge of simultaneously modeling global and local dependencies in remote sensing image super-resolution. They designed a global-detail dual-branch structure and introduced a dual-group multiscale wavelet loss constraint mechanism to enhance reconstruction fidelity. This method achieves superior performance on multiple benchmark datasets compared to existing approaches while significantly reducing computational complexity. This study provides new insights into the application of spatial-frequency-domain methods to infrared image super-resolution. To address the inherent limitations of infrared images, such as low signal-to-noise ratios and blurred details, self-supervised learning has shown significant promise. For instance, the transformer-based self-supervised blind spot denoising network proposed by Li et al.^60^ offers an efficient solution for denoising preprocessing in infrared images, thereby enhancing the outcomes of subsequent fusion tasks. In terms of feature representation, multi-granularity feature enhancement networks represent another effective strategy. For example, Guo et al.^61^ enhanced pedestrian features in both infrared and visible images using a multi-granularity feature enhancement network, successfully achieving cross-modal person re-identification.

These emerging methodologies, derived from fields such as image denoising and image enhancement, offer new theoretical perspectives and practical technical pathways for multimodal image fusion research.

## Methods

This section first outlines the framework of the fusion method, then details the training strategy and loss function.

### Fusion method architecture

**Fig. 1:**
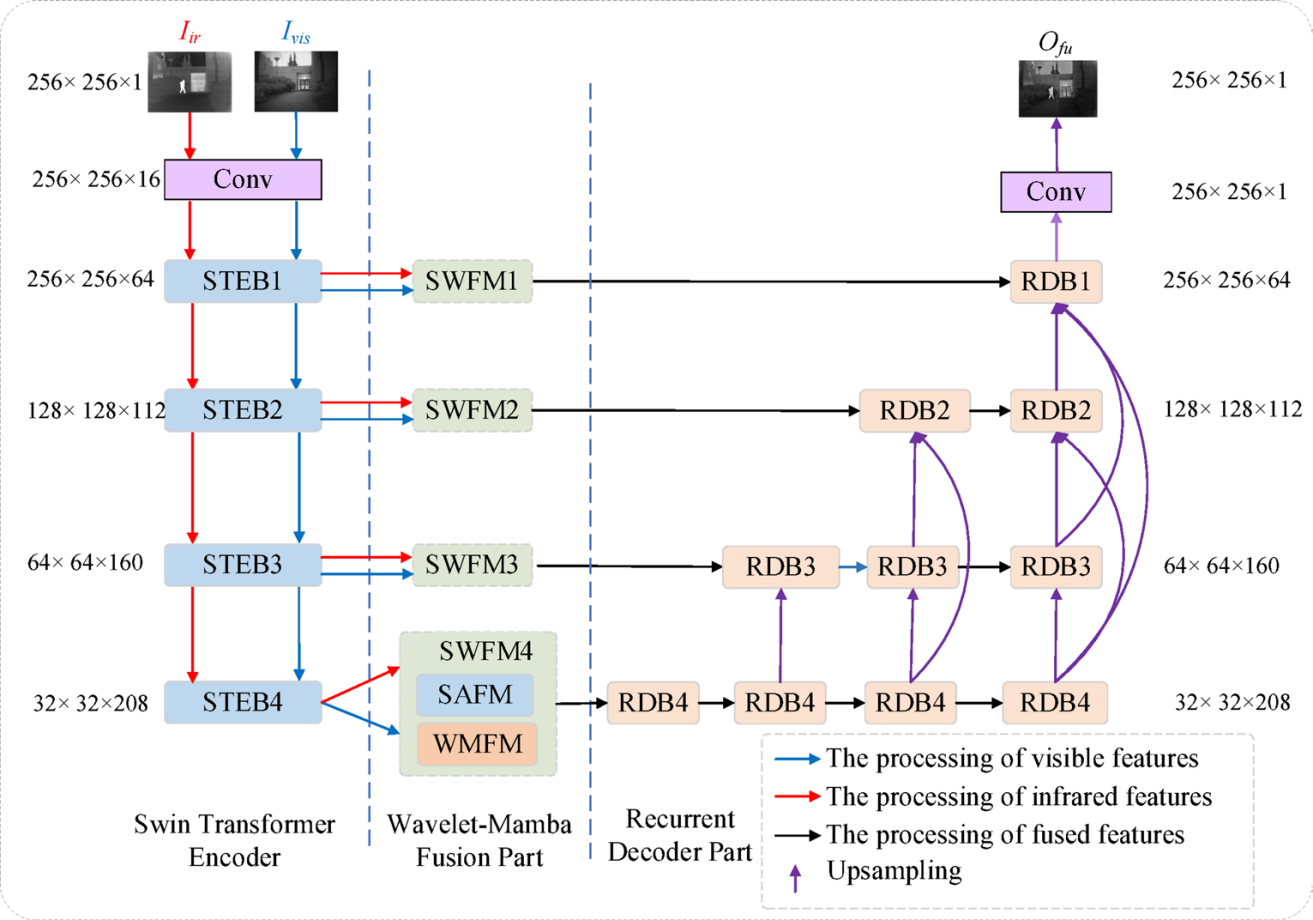
The architecture of the fusion method.

The framework of the proposed fusion method is illustrated in Fig. 1 and consists of three main components: the encoder, the fusion module, and the decoder. In Fig. 1, “*Iir*” and “*Ivis*” denote the infrared and visible source images, respectively. “*O*_*fu*_” represents the fused output image.

### Encoder part

**Fig. 2:**
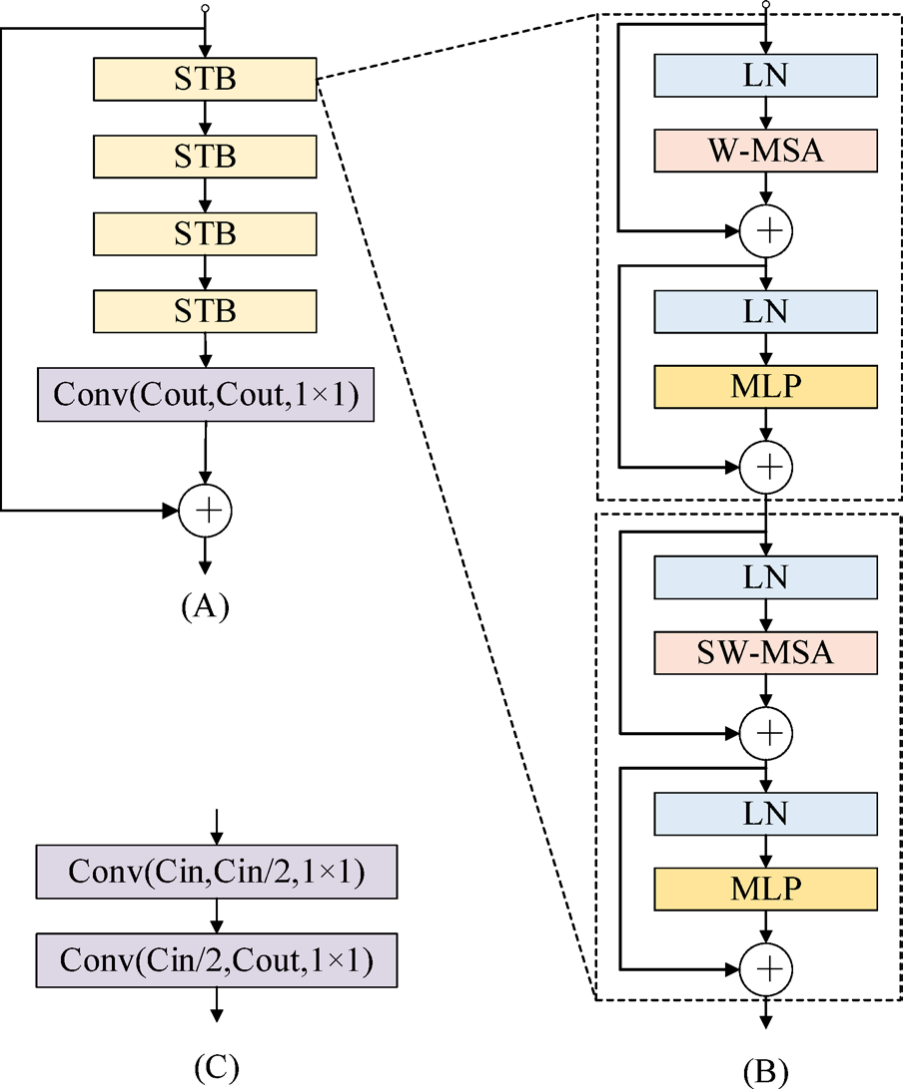
(**a**)The architecture of the Swin Transformer encoder block. (**b**) The architecture of the Swin Transformer block. (**c**) The architecture of the recurrent decoder block.

The encoder consists of a 1 × 1 convolutional layer followed by four Swin Transformer Encoder Blocks (STEBs). As shown in Fig. 2, each STEB comprises four Swin Transformer Blocks (STBs), with each STB based on the architecture proposed by Liu et al.^62^. Additionally, residual connections are incorporated to enhance feature representation. The mathematical description of the encoder is provided as follows.

${I}_{ir}$ and ${I}_{vis}$ represent the inputs of the infrared and visible source images, respectively. The deep features at the $l$-th level $(l=\mathrm{1,2},\mathrm{3,4})$, are computed as follows :

8 $$f_{x}^{1} = STEB_{1} \left( {Conv\left( {I_{x} } \right)} \right)$$

9 $$f_{x}^{l} = STEB_{l} \left( {f_{x}^{l - 1} } \right),{ }l = 2,3,4$$

Here $x=\{ir , vis\}$ represents the input feature maps, $Conv(\bullet )$ is the convolution operation, and ${STEB}_{l}\left(\bullet \right)$ is the Swin Transformer encoder block of $l$-th level. The channel dimensions of each layer are shown in Table 1.

**Table 1:** Channel dimension changes in each layer of the encoder.

Stage	Input channels	Output channels
Conv	1 (or 3)	16
STEB1	16	64
STEB2	64	112
STEB3	112	160
STEB4	160	208

### Decoder part

The decoder consists of four recurrent decoder blocks (RDBs), each containing two convolutional layers, as shown in Fig. 2c. A recurrent neural network is integrated into the convolutional neural network to reconstruct fused features across both spatial and temporal dimensions. Features at different levels are concatenated via dense connections, preserving both shallow and deep features in the output image and thereby enhancing image fusion performance.

The decoder’s mathematical formulation is as follows.

Stage 1:

10 $$O_{41} = RDB_{4} \left( {f_{fu}^{4} } \right)$$

Stage 2:

11 $$\begin{aligned} O_{42} = & RDB_{4} \left( {O_{41} } \right) \\ O_{32} = & RDB_{3} \left( {Conc\left( {f_{fu}^{3} ,O_{42} } \right)} \right) \\ \end{aligned}$$

Stage 3:

12 $$\begin{aligned} O_{43} = & RDB_{4} \left( {O_{42} } \right) \\ O_{33} = & RDB_{3} \left( {Conc\left( {O_{32} ,O_{43} } \right)} \right) \\ O_{23} = & RDB_{2} \left( {Conc\left( {f_{fu}^{2} ,O_{33} ,O_{43} } \right)} \right) \\ \end{aligned}$$

Stage 4:

13 $$\begin{gathered} O_{44} = RDB_{4} \left( {O_{43} } \right) \hfill \\ O_{34} = RDB_{3} \left( {Conc\left( {O_{33} ,O_{44} } \right)} \right) \hfill \\ O_{24} = RDB_{2} \left( {Conc\left( {O_{23} ,O_{34} ,O_{44} } \right)} \right) \hfill \\ O_{14} = RDB_{1} \left( {Conc\left( {f_{fu}^{1} ,O_{23} ,O_{34} ,O_{44} } \right)} \right) \hfill \\ \end{gathered}$$

where ${f}_{fu}^{l}$ represents the fused feature at $l$ level. $R{DB}_{p}$ denotes the recurrent decoder block of position $p$.

### Fusion part

**Fig. 3:**
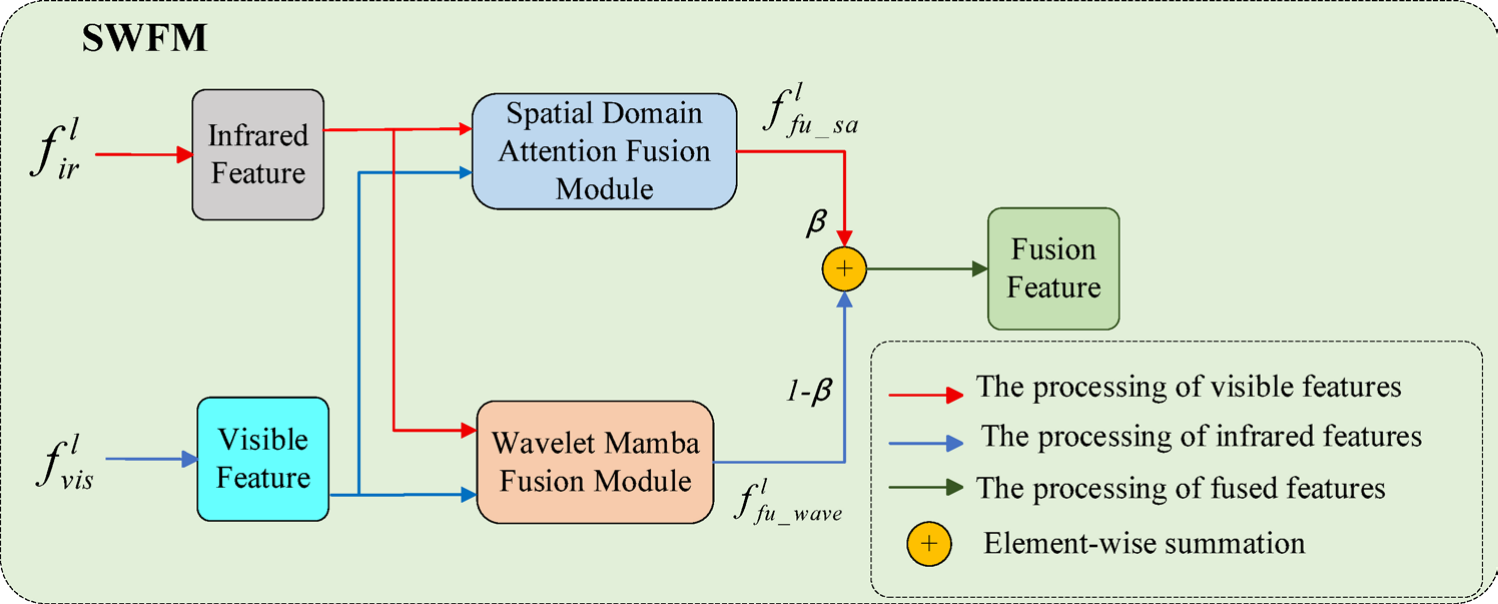
The architecture of the fusion network.

The learnable fusion networks (SWFM) are illustrated in Fig. 3. Each SWFM module comprises two branches: the spatial-domain attention fusion module (SAFM) and the Wavelet-Mamba fusion module (WMFM). The SAFM module performs spatial-domain feature fusion, whereas the WMFM module implements frequency-domain feature fusion via wavelet transforms and Mamba. Subsequently, the feature maps generated by SAFM and WMFM are combined using a weighted fusion strategy, expressed as follows:

14 $$f_{fu\_sa}^{l} = SAFM\left( {f_{ir}^{l} { },{ }f_{vis}^{l} } \right)$$

15 $$f_{fu\_wave}^{l} = WMFM\left( {f_{ir}^{l} { },{ }f_{vis}^{l} } \right)$$

16 $$f_{fu}^{l} = \beta \times f_{fu\_sa}^{l} { } + \left( {1 - \beta } \right) \times { }f_{fu\_wave}^{l}$$

where ${f}_{ir}^{l}\in {\mathbb{R}}^{C\times H\times W}$ denotes the infrared feature map, ${f}_{vis}^{l}\in {\mathbb{R}}^{C\times H\times W}$ represents the visible feature map. $SAFM(\bullet )$ and $WMFM(\bullet )$ refer to the spatial domain attention fusion module and wavelet mamba fusion module, respectively. The final fused feature map is denoted as ${f}_{fu}^{l}$, where ${f}_{fu}^{l}\in {\mathbb{R}}^{C\times H\times W}$, and $\beta$ is a learnable weight parameter.

**Fig. 4:**
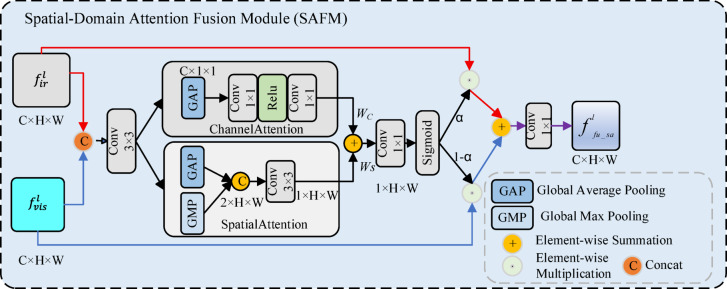
The architecture of the SAFM block.

*SAFM module* The SAFM module architecture is shown in Fig. 4. At its core is a parallel dual-path attention mechanism. First, the infrared and visible feature maps are concatenated along the channel dimension. This fused feature map is subsequently processed by a channel-spatial attention module, which consists of two parallel branches: one branch generates a channel attention weight vector ${W}_{c}$ ,which captures the relative importance of each channel. At the same time, the other produces a spatial attention weight matrix ${W}_{s}$ , which emphasizes salient regions. To fully leverage the complementary properties of channel and spatial attention, the two attention outputs are integrated and passed through a 1 × 1 convolution, followed by a Sigmoid activation, yielding a unified attention-enhanced feature map. Additionally, a skip connection is employed to reintroduce the original input features into the attention-weighted output. This design effectively mitigates the risk of gradient vanishing while facilitating efficient transmission of low-level information. Consequently, it not only enhances training stability but also strengthens the network’s representational capacity.

The overall process of this module is formulated as follows:

17 $$f_{ir\_vi}^{l} = Conv3 \times 3\left( {Concat\left( {f_{ir}^{l} f_{vis}^{l} } \right)} \right)$$

18 $$w_{c} = Conv1 \times 1\left( {Relu\left( {Conv1 \times 1\left( {GAP\left( {f_{ir\_vi}^{l} } \right)} \right)} \right)} \right)$$

19 $$w_{s} = Conv3 \times 3\left( {Concat(GAP\left( {f_{ir\_vi}^{l} } \right),GMP\left( {f_{ir\_vi}^{l} } \right)} \right))$$

20 $$f_{{{\mathrm{sa}}\_{\mathrm{attention}}}}^{l} = \sigma \left( {Conv1 \times 1\left( {w_{s} + w_{c} } \right)} \right)$$

21 $$f_{fu\_sa}^{l} = Conv1 \times 1(\alpha \times f_{sa\_attention}^{l} \times f_{ir}^{l} + { }\left( {1 - {\upalpha })f_{{{\mathrm{sa}}\_{\mathrm{attention}}}}^{l} \times f_{vis}^{l} } \right)$$

where $\alpha$ is a learnable parameter; $GAP\left(\bullet \right)$ and $GMP(\bullet )$ denote Global Average Pooling and Global Max Pooling, respectively; $\sigma$ denotes the Sigmoid function; and ReLU refers to the rectified linear unit activation function.

**Fig. 5:**
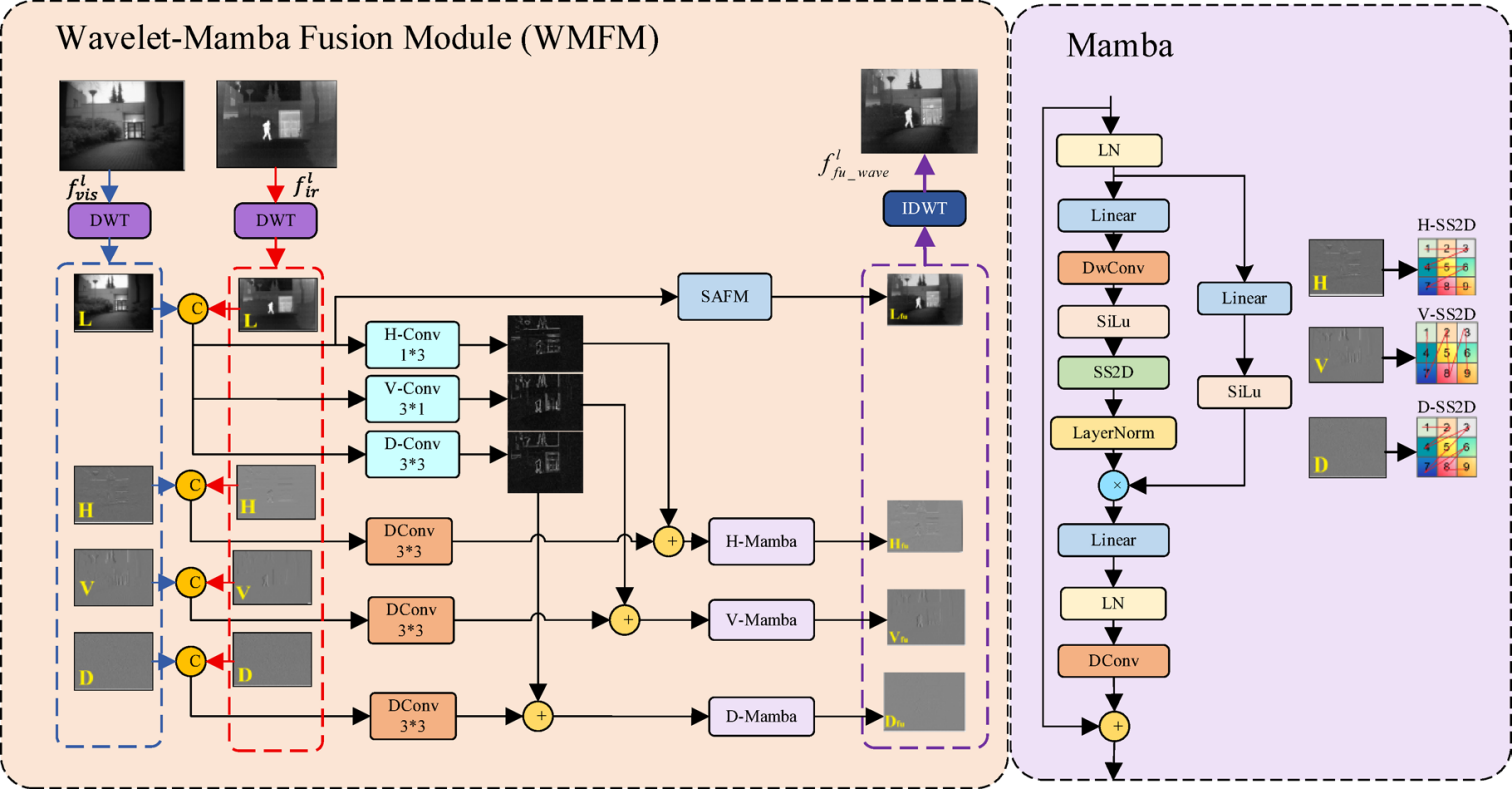
The architecture of the WMFM block.

*WMFM Module* This module employs wavelet decomposition (DWT) to decompose infrared and visible features into low-frequency (LF) and high-frequency (HF) components. The SAFM module fuses the low-frequency components, while the Mamba module enhances the high-frequency components. Finally, the enhanced high-frequency components and the fused low-frequency components are reconstructed through inverse wavelet transform (IWDT) to generate the final output. The overall workflow of this module is illustrated in Fig. 5.

The overall process of this module is formulated as follows:

First, low-frequency and high-frequency components of the visible and infrared feature maps are obtained using the Haar wavelet transform. The computation is expressed as follows:

22 $$\left\{ {\begin{array}{*{20}c} {{\mathrm{L}}_{ir} ,{\mathrm{H}}_{ir} {\text{, V}}_{ir} {, }D_{ir} {\text{ = DWT(}}f_{ir} {)}} \\ {{\text{ L}}_{vi} ,{\mathrm{H}}_{vi} {\text{, V}}_{vi} {, }D_{vi} {\text{ = DWT(}}f_{vis} {)}} \\ \end{array} } \right.$$

where ${f}_{ir},{f}_{vis}\in {\mathbb{R}}^{\mathrm{C}\times \mathrm{H}\times \mathrm{W}}$. ${L}_{ir/vi}$ denotes low-frequency information. ${H}_{ir/vi}$ *,*${V}_{ir/vi}$*, and* ${D}_{ir/vi}$ denote the high-frequency details along the horizontal, vertical, and diagonal directions of the feature maps. $DWT\left(\bullet \right)$ represents the Haar wavelet transform.

The low-frequency and high-frequency information is concatenated along the channel, and the SAFM module fuses the low-frequency information. Simultaneously, the horizontal, vertical, and diagonal features of the low-frequency information are extracted via *H_Conv*, *V_Conv*, and *D_Conv*, respectively. These features are then fused with the corresponding high-frequency details to enhance them. The calculation formula is as follows:

23 $$\left\{ \begin{gathered} L_{ir\_vi} = Concat\left( {L_{ir} ,L_{vi} } \right) \hfill \\ L_{fu} = SAFM\left( {L_{ir\_vi} } \right) \hfill \\ H_{fu} = H\_Mamba\left( {DConv3 \times \left( {3Concat\left( {H_{ir} ,H_{vi} } \right)} \right) + H\_Conv3 \times 1\left( {L_{ir\_vi} } \right)} \right) \hfill \\ V_{fu} = V\_Mamba\left( {DConv3 \times 3\left( {Concat\left( {V_{ir} ,V_{vi} } \right)} \right) + V\_Conv1 \times 3\left( {L_{ir\_vi} } \right)} \right) \hfill \\ D_{fu} = D\_Mamba\left( {DConv3 \times 3\left( {Concat\left( {D_{ir} ,D_{vi} } \right)} \right) + D\_Conv3 \times 3\left( {L_{ir\_vi} } \right)} \right) \hfill \\ \end{gathered} \right.$$

where $Concat\left(\bullet \right)$ denotes the Concatenation operation. *SAFM(.)* is the spatial domain attention fusion module. $DConv(\bullet )$ denotes the deep convolutional. $H\_Mamba(\bullet )$ represents the horizontally scanning Mamba module. $V\_Mamba(\bullet )$ represents the vertically scanning Mamba block. $D\_Mamba(\bullet )$ represents the diagonally scanning Mamba block.

 Finally, the combined low-frequency and high-frequency information is transformed back into the spatial domain using the inverse wavelet transform. The corresponding formulation is as follows:

24 $$f_{fu\_wave} {\text{ = IDWT}}\left( {L_{fu} {\text{, H}}_{fu} ,V_{fu} ,D_{fu} } \right)$$

The algorithm of the WMFM module is described as follows:

**Algorithm 1:**
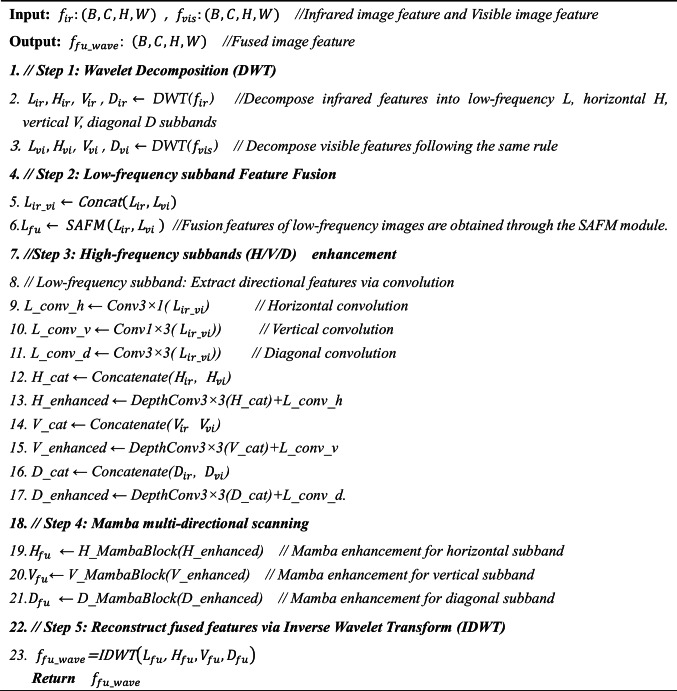
Wavelet mamba fusion module (WMFM).

### Training of the encoder-decoder network

**Fig. 6:**
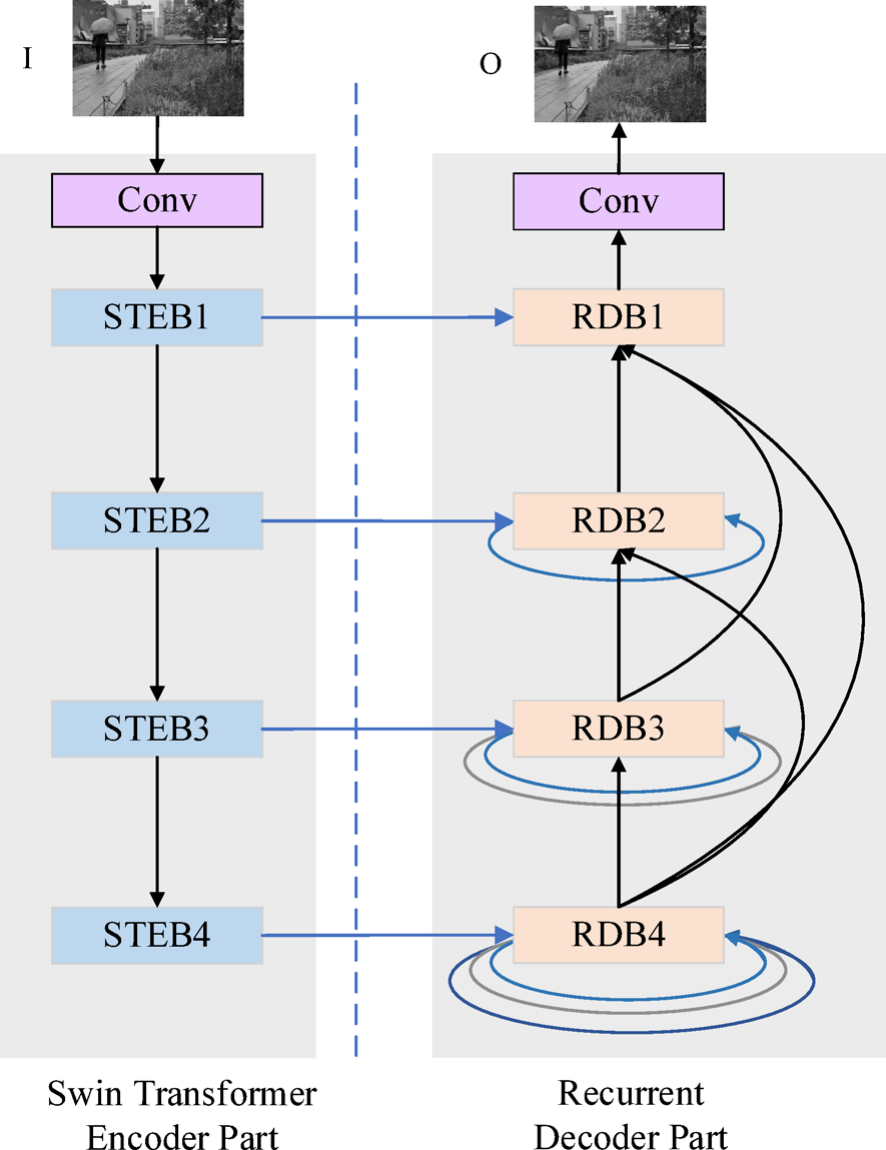
The architecture of the encoder-decoder network training process.

As illustrated in Fig. 6, the encoder-decoder network architecture for training consists solely of the encoder and decoder components. Here, “*I*” represents the input image, and “*O*” represents the output image.

The network is trained using a loss function ${L}_{coder}$ defined by (25).

25 $$L_{coder} = \lambda_{1} L_{mse} + \lambda_{2} L_{ssim}$$

where ${L}_{mse}$ denotes mean-squared loss , ${L}_{ssim}$ denotes the structure similarity loss. ${\lambda }_{1}$ and ${\lambda }_{2}$ are the trade-off parameters between ${L}_{mse}$ and ${L}_{ssim}$.

The mean-squared loss ${L}_{mse}$ is defined in (26).

26 $$L_{mse} = \|O - I\|_{2}^{2}$$

The structure similarity loss ${L}_{ssim}$ is defined in (27).

27 $$L_{ssim} = 1 - SSIM\left( {O,I} \right)$$

where $SSIM(\bullet ,\bullet )$ denotes the structural similarity index as introduced by Wang et al.^63^.

### Training of the fusion network

During this phase, the encoder-decoder network is frozen, and only the fusion network is trained.

To train the fusion network, the loss function ${L}_{fusion}$ as defined in (28).

28 $$L_{fusion} = L_{feature} + \lambda_{3} \tilde{L}_{ssim}$$

where ${L}_{feature}$ denotes the feature loss, and ${\widetilde{L}}_{ssim}$ represents the structural similarity loss between the source image and the output image. ${\lambda }_{3}$ is the trade-off parameter.

The feature loss ${L}_{feature}$ is defined in (29).

29 $$L_{feature} = \left\| {\nabla X_{fu} - \nabla X_{vis} } \right\|_{2}^{2} + \left\| {\nabla X_{fu} - \nabla X_{ir} } \right\|_{2}^{2}$$

where $\nabla$ denotes the Sobel gradient operator, ${X}_{fu}$ represents the fused image, ${X}_{vis}$ and ${X}_{ir}$ are the input visible and infrared image.

The structure similarity loss ${\widetilde{L}}_{ssim}$ is defined in (30).

30 $$\tilde{L}_{ssim} = \left( {1 - SSIM\left( {X_{fu} ,X_{vis} } \right)} \right) + \left( {1 - SSIM\left( {X_{fu} ,X_{ir} } \right)} \right)$$

## Experiments and result analysis

This section begins with training and testing, followed by ablation studies. Finally, a quantitative and qualitative comparative analysis is presented that evaluates our method against state-of-the-art algorithms.

### Training and testing details

In the first stage, the encoder-decoder is trained using the COCO dataset^64^. In the second stage, the fusion network is trained on the LLVIP dataset^65^, with the learning rate set to $1\times {10}^{-4}$ , and the batch size set to 4. λ₁ and λ₂ were set to 1 and 100. ${\lambda }_{3}$ was set to 200.

During the testing phase, 30 image pairs from the TNO dataset, 50 from the RoadScene dataset, and 360 from the MSRS dataset were selected, comprising images of military scenes, people, and street views.

Nine evaluation metrics were used to assess fusion performance: Information Entropy (EN) and Standard Deviation (SD), which measure total information content and image contrast, respectively. DCT (${\mathrm{FMI}}_{dct}$) and Wavelet Transform (${\mathrm{FMI}}_{wt}$) assess the degree of feature preservation. Visual Information Fidelity (VIF) assesses the amount of perceptual information from a human visual perspective, while Sum of Correlation of Differences (SCD) evaluates the ability to integrate complementary information. Average Gradient (AG) quantifies detail sharpness, and the fusion quality index (${Q}^{AB/F}$) focuses on the quality of edge information transfer. Finally, Structural Similarity (SSIM) comprehensively assesses structural fidelity across multiple scales. To ensure evaluation accuracy, the average of various assessments is used as the final result.

Our model is implemented using PyTorch and trained on an NVIDIA RTX 3090 graphics card.

### Ablation study

This section employs systematic ablation experiments to evaluate the contributions of the Swin Transformer encoder block (STEB), the recurrent decoding block (RDB), and the fusion network (SWFM) to the model’s performance. Four experimental configurations are designed for comparative analysis: ① replacing the STEB module with convolutional blocks in the encoder (Proposed w/o STEB); ②substituting the RDB module with a nested architecture in the decoder (Proposed w/o RDB); ③ replacing the SWFM fusion method with a weighted average strategy (Proposed w/o SWFM); and ④ including all components proposed in this work(Proposed).

**Fig. 7:**
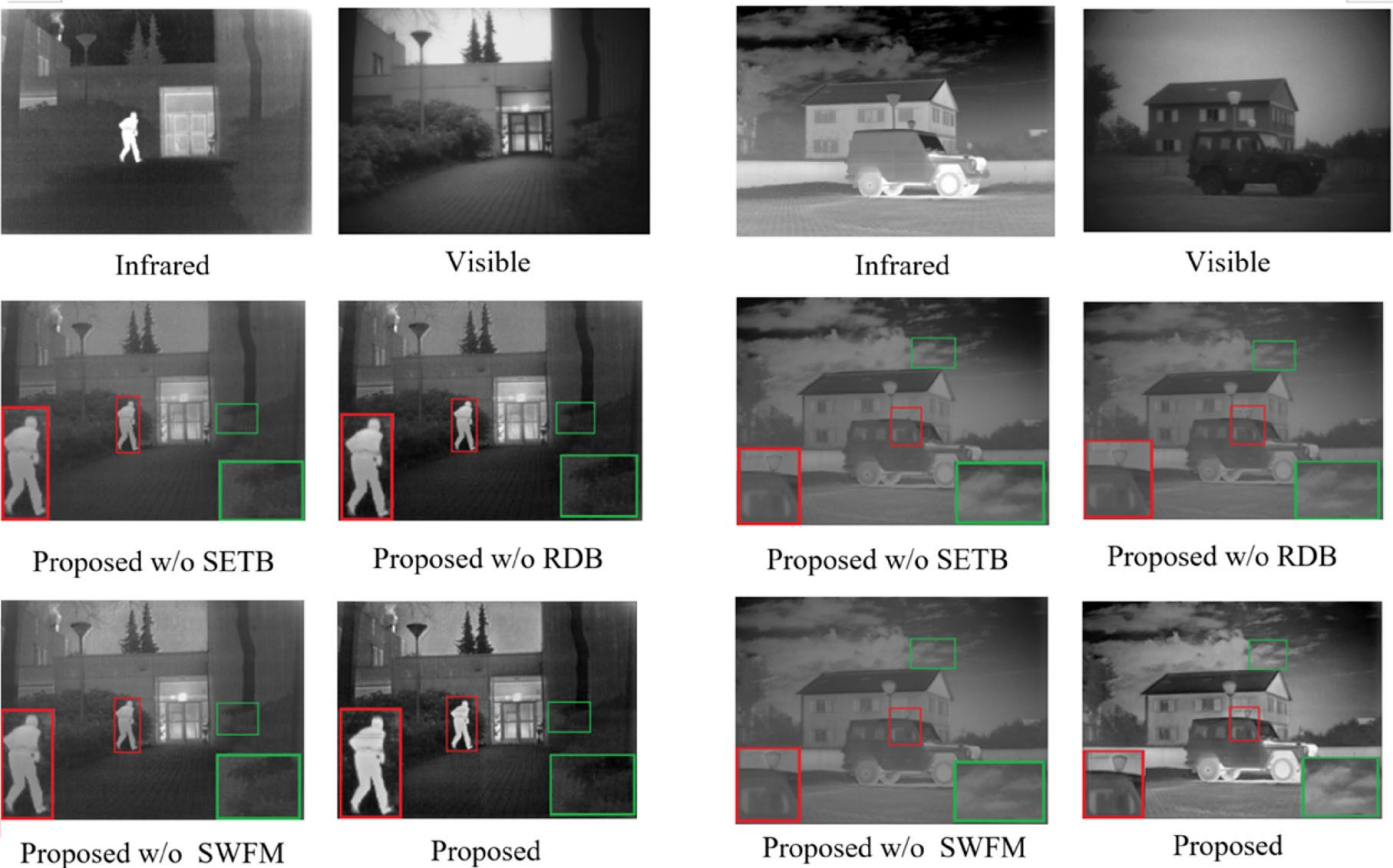
Subjective results of ablation experiments on the TNO database. The source images are from https://figshare.com/articles/dataset/TNO_Image_Fusion_Dataset/1008029. All fusion results are generated by the authors using the proposed method.

The subjective evaluation results for the four configurations on the TNO dataset are shown in Fig. 7. The green boxes highlight detailed texture features, while the red boxes denote thermal radiation characteristics. The “Proposed w/o STEB” and “w/o RDB” configurations exhibit more visible image features but lack sufficient infrared image characteristics and clear edge definition. For instance, in Fig. 7, while the shrub details within the green boxes are well-preserved, the target edges in the red boxes are not sufficiently sharpened. In the absence of the Spatial Attention Fusion Module and the Wavelet Mamba Fusion Module, the “Proposed w/o SWFM” configuration fails to properly retain both infrared and visible features during fusion. For example, the visible image’s window scene features are lost within the red box, while the cloud characteristics in the infrared image are missing within the green box. In contrast, the entire configuration effectively preserves both thermal radiation information from infrared images and detailed texture information from visible images, ensuring clear target visibility and sharp texture definition.

The objective experimental results, summarized in Table 2, highlight the superiority of the proposed network over the “Proposed w/o STEB” and “Proposed w/o RDB” networks across several metrics, including VIF, SCD, AG, ${\mathrm{FMI}}_{dct}$, and SSIM. The proposed network also secures the second-best performance in EN, SD and ${Q}^{AB/F}$. This remarkable performance can be attributed to the inclusion of Swin Transformer blocks in the feature-extraction process and to the use of a recurrent architecture in the image-reconstruction stage. These components work synergistically to introduce redundancy into the fusion output, thereby enhancing the network’s overall quality and effectiveness. In contrast, although the “Proposed w/o SWFM” variant shows a slight advantage in metrics like EN, SD, and ${Q}^{AB/F}$, it performs significantly worse in other critical metrics. This suggests that while directly summing infrared and visible features with equal weights may marginally improve the information content of the fused image, it fails to accurately capture the essential features and structures of the source images. Consequently, this demonstrates that the traditional weighted averaging method is unsuitable for complex tasks that involve fusing infrared and visible images. This finding aligns with the subjective evaluation results.

**Table 2:** Objective experimental results of the ablation study. The best values are bold, the second-best values are underlined, and the third-best values are italic.

Model	EN↑	SD↑	${\mathbf{F}\mathbf{M}\mathbf{I}}_{{\boldsymbol{d}}{\boldsymbol{c}}{\boldsymbol{t}}}\uparrow$	${\mathbf{F}\mathbf{M}\mathbf{I}}_{{\boldsymbol{w}}{\boldsymbol{t}}}\uparrow$	VIF↑	SCD↑	${{\boldsymbol{Q}}}^{{\boldsymbol{A}}{\boldsymbol{B}}/{\boldsymbol{F}}}$↑	AG↑	SSIM↑
Proposed w/o STEB	*6.906*	*38.357*	0.904	**36.032**	*0.652*	1.834	*0.425*	*4.562*	0.670
Proposed w/o RDB	6.862	37.248	*0.852*	35.022	0.663	*1.817*	0.424	4.321	*0.693*
Proposed w/o SWFM	**7.086**	**40.378**	0.829	33.531	0.645	1.695	**0.489**	4.892	0.705
Proposed	6.992	38.959	**1.032**	*34.949*	**0.686**	**1.844**	0.516	**5.151**	**0.718**

The effectiveness of STEDs, RDBs, and SWFMs is evaluated through both subjective and objective assessments. Incorporating STEDs and RDBs into the encoder-decoder network improves its ability to extract and retain features. Meanwhile, SWFMs autonomously balance infrared and visible features during feature extraction, thereby improving fusion performance.

### Fusion results analysis

We select nine state-of-the-art fusion algorithms for comparison with our proposed method. These include two AE-based algorithms (NestFuse^36^, Res2Fusion^38^), two CNN-based algorithms (U2Fusion^18^, CrossFuse^27^), two transformer-based algorithms (SwinFusion^25^, ATFusion^28^), a GAN-based algorithm (FusionGAN^21^), and two mamba-based algorithms (FusionMamba^34^, MambaDFuse^47^).

**Fig. 8:**
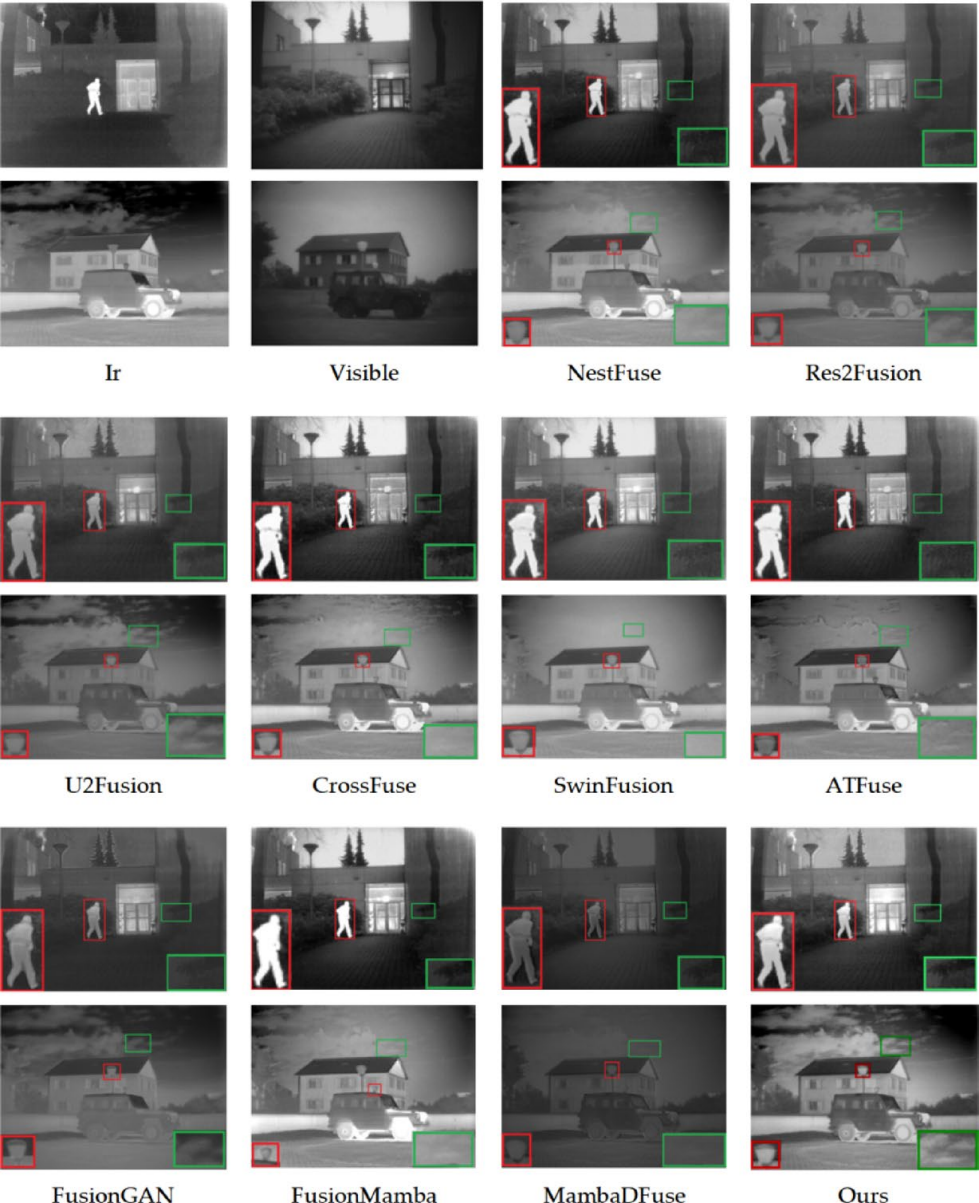
Subjective experimental results for the TNO dataset. The source images are from the TNO dataset. All fusion results (including those of comparison methods) are generated by the authors using publicly available code or reproductions based on the original papers to ensure fair comparison.

**Fig. 9:**
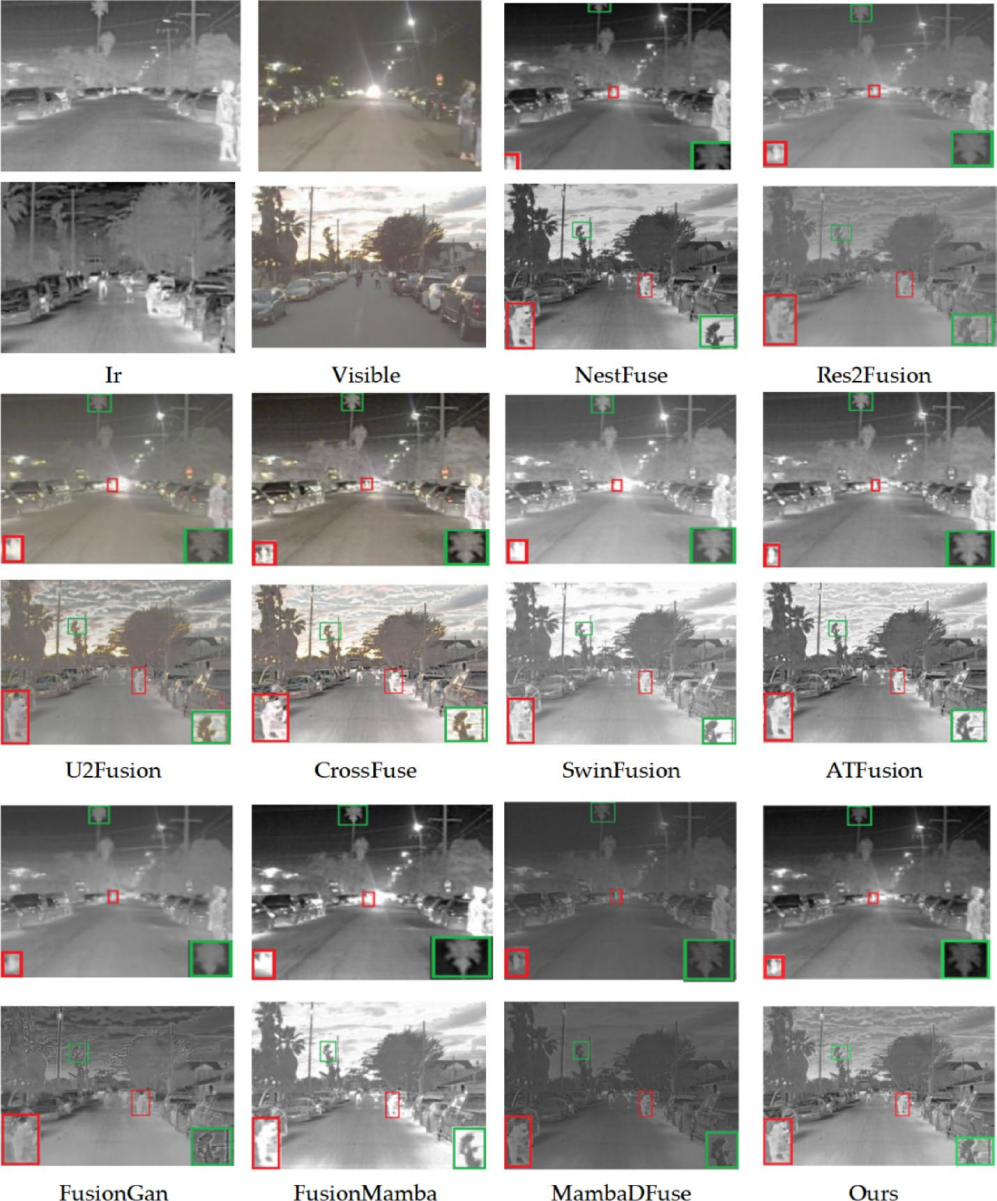
Subjective experimental results for the Road dataset. The source images are from https://github.com/hanna-xu/RoadScene. All fusion results (including those of comparison methods) are generated by the authors using publicly available code or reproductions based on the original papers to ensure fair comparison.

**Fig. 10:**
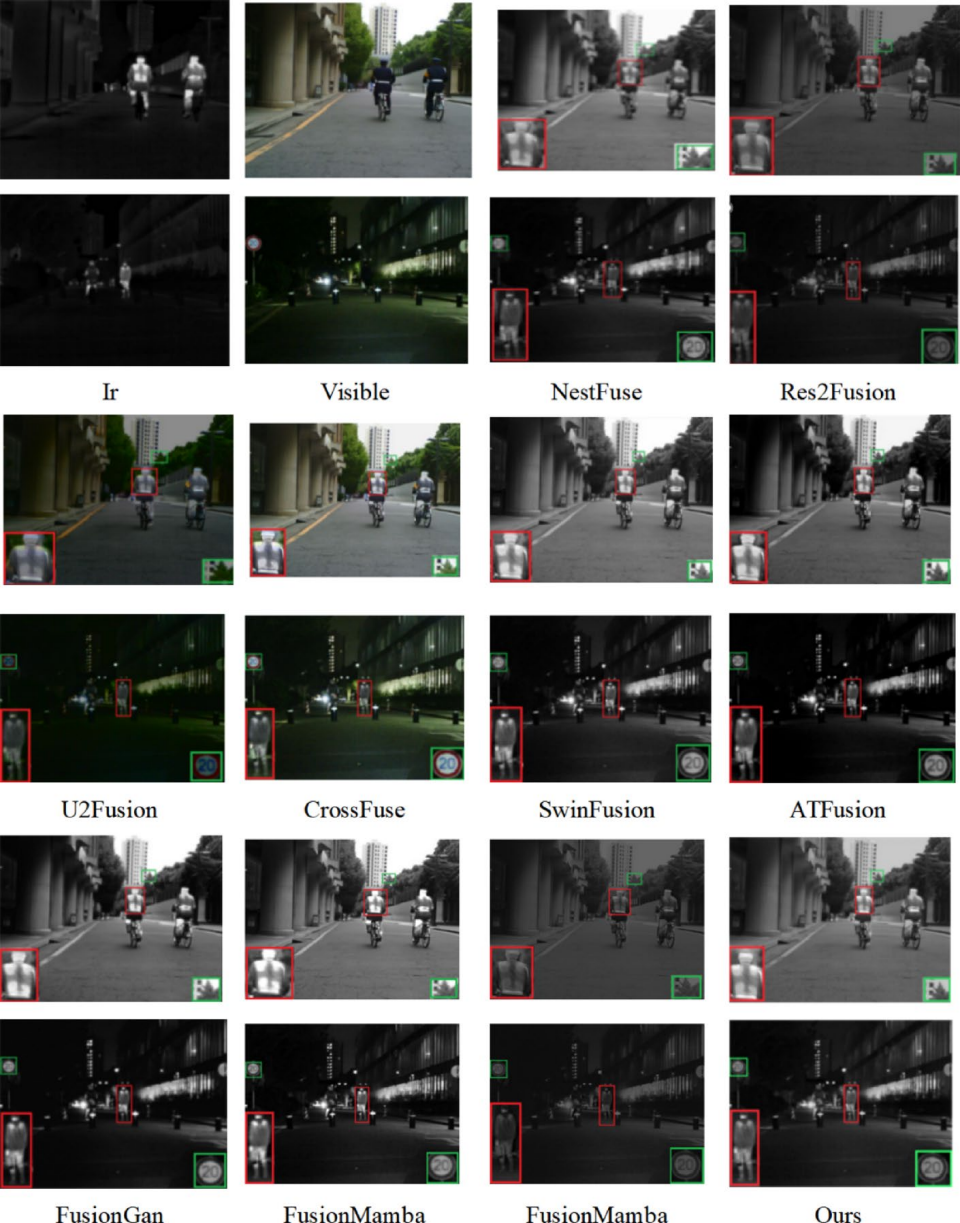
Subjective experimental results for the MSRS dataset. The source images are from https://github.com/Linfeng-Tang/MSRS. All fusion results (including those of comparison methods) are generated by the authors using publicly available code or reproductions based on the original papers to ensure fair comparison.

The subjective evaluation results, presented in Figs. 8, 9, 10, include verification for both the TNO, RoadScene, and MSRS datasets. The fusion results, presented in three image groups, highlight the algorithms’ varying performance. The fusion outputs of Res2Fusion, U2Fusion, and MambaDFuse exhibit low contrast with the targets and fail to retain thermal radiation information, as indicated by the red boxes in Fig. 8. Although methods such as CrossFuse, SwinFusion, ATFuse, and FusionMamba exhibit better contrast performance, they suffer from blurred boundaries, as evidenced by the loss of cloud detail and the indistinct window outlines in Fig. 8. Additionally, targets under intense illumination are adversely affected, as evidenced by the blurred pedestrians in Fig. 9.

The quantitative analysis results of the algorithm on TNO, RoadScene, and MSRS datasets are presented in Tables 3, 4, 5. Our method outperforms all other algorithms across four key metrics: ${\mathrm{FMI}}_{dct}$, SCD, AG, VIF, and SSIM. These results demonstrate the method’s balanced performance across all metrics, without significant weaknesses. The superior SCD and SSIM scores indicate that WMambaFuse effectively preserves source-image information with minimal distortion, a result attributed to the Swin Transformer encoder and the “WMFM” and “SAFM” fusion blocks.

**Table 3:** Objective experimental results of the fusion process on TNO.

Model	EN↑	SD↑	${\mathbf{F}\mathbf{M}\mathbf{I}}_{{\boldsymbol{d}}{\boldsymbol{c}}{\boldsymbol{t}}}$↑	${\mathbf{F}\mathbf{M}\mathbf{I}}_{{\boldsymbol{w}}{\boldsymbol{t}}}\uparrow$	VIF↑	SCD↑	${{\boldsymbol{Q}}}^{{\boldsymbol{A}}{\boldsymbol{B}}/{\boldsymbol{F}}}$↑	AG↑	SSIM↑
NestFuse	*7.045*	*42.032*	0.791	21.553	**0.692**	1.698	0.486	3.726	0.677
Res2Fusion	6.291	23.777	0.775	21.241	0.472	1.561	0.348	2.398	0.707
U2Fusion	6.261	29.631	0.571	13.819	0.562	0.552	0.107	2.321	0.449
CrossFuse	**7.161**	47.372	0.885	23.631	*0.652*	1.685	0.516	5.151	0.655
SwinFusion	6.809	37.856	*0.863*	*24.855*	0.645	*1.691*	*0.509*	3.974	*0.702*
ATFusion	6.951	39.666	0.803	20.297	0.607	1.599	**0.575**	*4.198*	0.682
FusionGAN	6.326	25.465	0.841	26.327	0.485	1.531	0.354	2.803	0.694
MambaDFuse	5.497	13.317	0.802	19.516	0.382	1.08	0.209	2.063	0.667
FusionMamba	7.092	**47.392**	0.736	17.678	0.474	1.612	0.337	3.203	0.654
Proposed	6.992	38.959	**0.931**	**34.949**	0.686	**1.844**	0.446	**5.637**	**0.718**

The best values are bold, the second-best values are underlined, and the third-best values are italic.

**Table 4:** Objective experimental results of the fusion process on RoadScene.

Model	EN↑	SD↑	${\mathbf{F}\mathbf{M}\mathbf{I}}_{{\boldsymbol{d}}{\boldsymbol{c}}{\boldsymbol{t}}}$↑	${\mathbf{F}\mathbf{M}\mathbf{I}}_{{\boldsymbol{w}}{\boldsymbol{t}}}$↑	VIF↑	SCD↑	${{\boldsymbol{Q}}}^{{\boldsymbol{A}}{\boldsymbol{B}}/{\boldsymbol{F}}}$↑	AG↑	SSIM↑
NestFuse	*7.287*	47.504	0.732	21.003	0.720	1.714	*0.553*	4.521	0.667
Res2Fusion	6.637	28.477	0.699	20.523	0.520	1.414	0.384	2.883	*0.702*
U2Fusion	6.816	32.344	0.574	17.045	0.626	0.363	0.114	2.912	0.411
CrossFuse	**7.390**	50.191	0.717	20.318	**0.765**	1.679	0.596	*5.694*	0.667
SwinFusion	7.039	44.997	*0.769*	*22.814*	0.677	*1.713*	0.503	4.269	**0.713**
ATFusion	7.315	*47.741*	0.719	20.267	0.625	1.584	**0.658**	*5.313*	0.674
FusionGAN	6.855	35.736	0.781	25.676	0.532	1.478	0.306	2.751	0.682
MambaDFuse	6.021	19.083	0.701	20.214	0.432	0.868	0.292	2.675	0.664
FusionMamba	7.205	**56.418**	0.708	20.217	0.592	1.647	0.417	4.448	0.659
Proposed	7.015	37.107	**0.852**	**33.041**	*0.702*	**1.733**	0.476	**5.847**	0.706

The best values are bold, the second-best values are underlined, and the third-best values are italic.

**Table 5:** Objective experimental results of the fusion process on MSRS.

Model	EN↑	SD↑	${\mathbf{F}\mathbf{M}\mathbf{I}}_{{\boldsymbol{d}}{\boldsymbol{c}}{\boldsymbol{t}}}$↑	${\mathbf{F}\mathbf{M}\mathbf{I}}_{{\boldsymbol{w}}{\boldsymbol{t}}}\uparrow$	VIF↑	SCD↑	${{\boldsymbol{Q}}}^{{\boldsymbol{A}}{\boldsymbol{B}}/{\boldsymbol{F}}}$↑	AG↑	SSIM↑
NestFuse	**6.984**	*47.042*	0.751	18.849	0.759	1.456	0.384	*3.027*	0.662
Res2Fusion	6.311	27.501	0.819	21.204	0.601	1.262	0.374	2.395	**0.697**
U2Fusion	5.682	29.595	0.682	15.348	0.652	1.373	0.323	2.297	0.671
CrossFuse	6.858	47.492	*0.854*	24.071	0.782	1.674	*0.627*	4.449	0.667
SwinFusion	*6.822*	46.874	0.935	28.166	*0.762*	**1.681**	0.664	3.962	0.693
ATFusion	6.653	**48.098**	0.848	*24.978*	0.695	*1.648*	**0.679**	*4.091*	0.585
FusionGAN	5.083	17.543	0.702	19.437	0.601	1.006	0.207	1.852	0.645
MambaDFuse	5.704	18.304	0.798	19.647	0.503	0.787	0.228	1.878	0.665
FusionMamba	6.781	46.882	0.723	18.312	0.667	1.634	0.427	3.025	*0.687*
Proposed	6.506	35.129	**0.952**	**34.259**	**0.824**	1.500	0.557	**5.321**	0.655

The best values are bold, the second-best values are underlined, and the third-best values are italic.

**Fig. 11:**
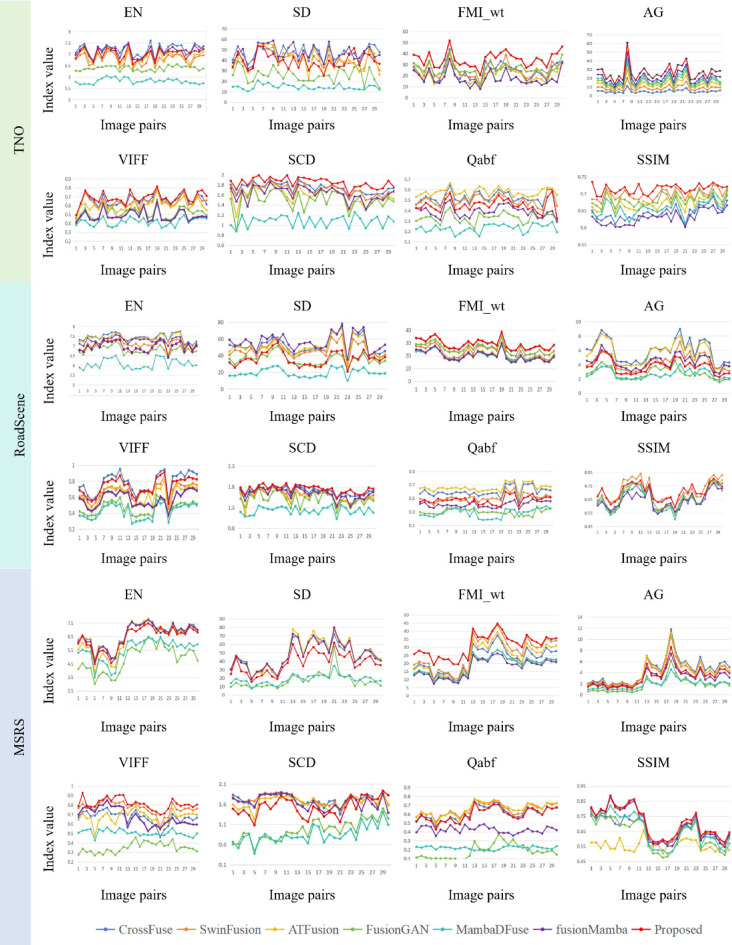
Quantitative comparison results of our method on TNO, RoadScene, and MSRS datasets. Our method is shown in red.

To ensure a more comprehensive quantitative evaluation, we randomly selected 30 image pairs from each of the three datasets and conducted individual performance comparisons with six recently high-performing algorithms. The results are shown in Fig. 11. As shown in the figure, the performance of the same algorithm varies across different image pairs, demonstrating the effectiveness and rationality of using diverse, large-scale datasets.

### Complexity and efficiency analysis of the fusion model

To quantitatively evaluate the operational efficiency of the proposed fusion model, we conducted a comparative analysis with mainstream fusion methods across three dimensions: the number of parameters (Parameters/M), floating-point operations (FLOPs/G), and inference time (Time/s). Inference time refers to the average processing time per image, calculated on the MSRS test set (with an image resolution of 640 × 480). The detailed comparison results are presented in Table 6.

**Table 6:** Comparison of complexity and efficiency among different fusion models.

Method	Net	Flops(G)	Parameters(M)	Time(s)
AE	DenseFuse	22.71	0.89	0.23
	Res2Fusion	30.04	0.42	0.38
CNN	U2Fusion	201.36	2.64	0.22
GAN	FreqGAN	33.21	1.83	0.41
TransFomer	CrossFuse	142.35	21.03	0.16
	SwinFusion	276.67	0.93	3.29
Mamba	MambaDFuse	18.24	5.43	0.26
	FusionMamba	661.85	220.93	0.25
	Proposed	53.39	4.51	0.33

The proposed model achieves a favorable balance among FLOPs, parameter count, and inference time, demonstrating clear advantages over most existing Transformer and Mamba-based models in terms of computational complexity and model size. Nevertheless, incorporating Swin Transformer modules into the encoder introduces some computational overhead, leaving room for further optimization to improve inference speed. In future work, we will explore more efficient lightweight design strategies to better balance model performance and efficiency while maintaining fusion quality.

## Conclusions

This paper proposes a novel method for infrared and visible image fusion that integrates the Swin Transformer architecture, recurrent networks, and a “spatial-frequency domain” fusion strategy. This architecture enhances feature extraction capabilities while preserving more feature information in the fusion results. Furthermore, a learnable fusion network based on the spatial domain and small-Mamba is employed to leverage complementary advantages of local details and global structural information, thereby enabling robust feature fusion. Finally, the effectiveness of the proposed method is demonstrated through subjective and objective evaluations on publicly available datasets.

Although this method provides a novel and effective approach for infrared and visible image fusion, it still has certain limitations. While it performs well on public datasets, its generalizability under extreme imaging conditions such as dense fog or heavy rain remains to be validated. Future work will focus on exploring more robust fusion models. Additionally, image enhancement techniques for nighttime scenarios will be introduced to more effectively highlight key targets in infrared–visible image fusion.

## Data Availability

The infrared and visible image fusion datasets utilized in this study are publicly available resources, including MS-COCO ([https://cocodataset.org/] (https:/cocodataset.org)), LLVIP (https://bupt-ai-cz.github.io/LLVIP/), RoadScene(https://github.com/jiayi-ma/RoadScene), and TNO(https://figshare.com/articles/dataset/ TNO\_Image\_Fusion\_Dataset/1008029), and MSRS(https://github.com/Linfeng-Tang/MSRS).

## References

[1] Muller, A. C. & Narayanan, S. Cognitively-engineered multisensor image fusion for military applications. *Inf. Fusion* **10**(1), 137–149. 10.1016/j.inffus.2008.08.008 (2009).

[2] Liu, N., Li, W., Sun, X., Tao, R. & Chanussot, J. Remote sensing image fusion with task-inspired multiscale nonlocal-attention network. *IEEE Geosci. Remote Sens. Lett.* **20**, 1–5. 10.1109/LGRS.2023.3254049 (2023).

[3] Zhong, Y. et al. Unsupervised fusion of misaligned PAT and MRI images via mutually reinforcing cross-modality image generation and registration. *IEEE Trans. Med. Imaging* **43**, 1702–1714. 10.1109/TMI.2023.3347511 (2024). 38147426 10.1109/TMI.2023.3347511

[4] Zhang, X., Wang, X., Yan, C. & Sun, Q. EV-Fusion: A novel infrared and low-light color visible image fusion network integrating unsupervised visible image enhancement. *IEEE Sens. J.* **24**(4), 4920–4934. 10.1109/JSEN.2023.3346886 (2024).

[5] Fu, H., Sun, G., Ren, J., Zhang, A. & Jia, X. Fusion of PCA and segmented-PCA domain multiscale 2-D-SSA for effective spectral-spatial feature extraction and data classification in hyperspectral imagery. *IEEE Trans. Geosci. Remote Sens.* **60**, 1–14. 10.1109/TGRS.2020.3034655 (2022).

[6] Liu, J. et al. Patch-aware deep hyperspectral and multispectral image fusion by unfolding subspace-based optimization model. *IEEE J. Sel. Top. Appl. Earth Obs. Remote Sens.* **15**, 1024–1038. 10.1109/JSTARS.2022.3140211 (2022).

[7] Jian, L. et al. Infrared and visible image fusion based on deep decomposition network and saliency analysis. *IEEE Trans. Multimed.* **24**, 3314–3326. 10.1109/TMM.2021.3096088 (2022).

[8] Wang, X. et al. Contrast saliency information guided infrared and visible image fusion. *IEEE Trans. Comput. Imaging* **9**, 769–780. 10.1109/TCI.2023.3304471 (2023).

[9] Liu, L. et al. Median robust extended local binary pattern for texture classification. *IEEE Trans. Image Process.* **25**, 1368–1381. 10.1109/TIP.2016.2522378 (2016). 26829791 10.1109/TIP.2016.2522378

[10] Oral, M., & Turgut, S. S. A comparative study for image fusion. In *2018 Innovations in Intelligent Systems and Applications Conference (ASYU)*, 1–6, (2018). 10.1109/ASYU.2018.8554000

[11] Yin, H. & Xiao, J. Laplacian pyramid generative adversarial network for infrared and visible image fusion. *IEEE Signal Process. Lett.* **29**, 1988–1992. 10.1109/LSP.2022.3207621 (2022).

[12] Lei, Y. Research on image fusion algorithm based on wavelet transform. In *2023 IEEE 3rd International Conference on Electronic Technology, Communication and Information (ICETCI)*, 319–324, (2023). 10.1109/ICETCI57876.2023.10176556

[13] Jayashree, S., Karki, M. V., Indira, K., & Shivashankarappa, N. Performance analysis of image fusion rules implemented on nonsubsampled contourlet transform decomposed images for pansharpening. In *2022 4th International Conference on Circuits, Control, Communication and Computing (I4C)*, 103–108, (2022). 10.1109/I4C57141.2022.10057727

[14] Feng, X., Fang, C., Lou, X. & Hu, K. Research on infrared and visible image fusion based on tetrolet transform and convolution sparse representation. *IEEE Access* **9**, 23498–23510. 10.1109/ACCESS.2021.3056888 (2022).

[15] Veshki, F. G., & Vorobyov, S. A. Convolutional simultaneous sparse approximation with applications to RGB-NIR image fusion. *In 2022 56th Asilomar Conference on Signals, Systems, and Computers*, 872–876, (2022). 10.1109/IEEECONF56349.2022.10052057

[16] Zhang, Y., Zhang, L., Song, R., Huang, C. & Tong, Q. Considering nonoverlapped bands construction: A general dictionary learning framework for hyperspectral and multispectral image fusion. *IEEE Trans. Geosci. Remote Sens.* **61**, 1–15. 10.1109/TGRS.2023.3254555 (2023).

[17] Lai, J., Geng, J., Deng, X. & Jiang, W. DDFN: Deblurring dictionary encoding fusion network for infrared and visible image object detection. *IEEE Geosci. Remote Sens. Lett.* **20**, 1–5. 10.1109/LGRS.2023.3311176 (2023).

[18] Xu, H., Ma, J., Jiang, J., Guo, X. & Ling, H. U2Fusion: A unified unsupervised image fusion network. *IEEE Trans. Pattern Anal. Mach. Intell.* **44**(1), 502–518. 10.1109/TPAMI.2020.3012548 (2022). 32750838 10.1109/TPAMI.2020.3012548

[19] Tang, W., He, F., Liu, Y., Duan, Y. & Si, T. DATFuse: Infrared and visible image fusion via dual attention transform. *IEEE Trans. Circuits Syst. Video Technol.* **33**(6), 3159–3172. 10.1109/TCSVT.2023.3234340 (2023).

[20] Zhu, D., Ma, J., Li, D. & Wang, X. SCGAFusion: A skip-connecting group convolutional attention network for infrared and visible image fusion. *Appl. Soft Comput.* **163**, 111902. 10.1016/j.asoc.2024.111902 (2024).

[21] Ma, J. et al. FusionGAN: A generative adversarial network for infrared and visible image fusion. *Inf. Fusion* **48**, 11–26. 10.1016/j.inffus.2018.09.004 (2019).

[22] Ma, J. et al. DDcGAN: A dual-discriminator conditional generative adversarial network for multi-resolution image fusion. *IEEE Trans. Image Process.* **29**, 4980–4995. 10.1109/TIP.2020.2977573 (2020). 10.1109/TIP.2020.2977573 32167894

[23] Li, J., Huo, H., Li, C., Wang, R. & Feng, Q. AttentionFGAN: Infrared and visible image fusion using attention-based generative adversarial networks. *IEEE Trans. Multimed.* **23**, 1383–1396. 10.1109/TMM.2020.2997127 (2021).

[24] Chang, L. et al. DUGAN: Infrared and visible image fusion based on dual fusion paths and a U-type discriminator. *Neurocomputing* **578**, 127391. 10.1016/j.neucom.2024.127391 (2024).

[25] Ma, J. et al. SwinFusion: Cross-domain long-range learning for general image fusion via swin transformer. *IEEE/CAA J. Autom. Sin.* **9**(7), 1200–1217. 10.1109/JAS.2022.105686 (2022).

[26] Zhao, Z., Bai, H., Zhang, J., et al. CDDFuse: Correlation-driven dual-branch feature decomposition for multi-modality image fusion. (2023). 10.48550/arXiv.2211.14461

[27] Li, H. & Wu, X. J. CrossFuse: A novel cross attention mechanism-based infrared and visible image fusion approach. *Inf. Fusion* **103**, 102147. 10.1016/j.inffus.2023.102147 (2024).

[28] Yan, H., Xiong, S., Wang, L., et al. ATFusion: An alternate cross-attention transformer network for infrared and visible image fusion. Preprint at https://arxiv.org/abs/2401.11675. (2024).

[29] Gu, A., & Dao, T. Mamba: Linear-time sequence modeling with selective state spaces. (2023). 10.48550/arXiv.2312.00752

[30] Zhu, L., Liao, B., Zhang, Q., et al. Vision Mamba: Efficient visual representation learning with bidirectional state space model. Preprint at https://arxiv.org/pdf/2401.09417. (2024)

[31] Liu, Y., Tian, Y., Zhao, Y., et al. VMamba: Visual state space model. In *Proceedings of the 38th International Conference on Neural Information Processing Systems (NIPS’24)*, 103031–103062. 10.48550/arXiv.2401.10166. (2024).

[32] Huang, T., et al. LocalMamba: Visual state space model with windowed selective scan. (2024). 10.48550/arXiv.2403.09338

[33] Guo, H. et al. MambaIR: A simple baseline for image restoration with state-space model. *Lect. Notes Comput. Sci.* **15076**, 222–241. 10.1007/978-3-031-72649-1_13 (2025).

[34] Xie, X. et al. FusionMamba: Dynamic feature enhancement for multimodal image fusion with Mamba. *J. Intell. Fuzzy Syst.* **1**(1), 1–18. 10.1007/s44267-024-00072-9 (2024).

[35] Li, H. & Wu, X. J. DenseFuse: A fusion approach to infrared and visible images. *IEEE Trans. Image Process.* **28**(5), 2614–2623. 10.1109/TIP.2018.2887342 (2019). 10.1109/TIP.2018.2887342 30575534

[36] Li, H., Wu, X. J. & Durrani, T. NestFuse: An infrared and visible image fusion architecture based on nest connection and spatial/channel attention models. *IEEE Trans. Instrum. Meas.* **69**(12), 9645–9656. 10.1109/TIM.2020.3005230 (2020).

[37] Gao, S. H. et al. Res2Net: A new multi-scale backbone architecture. *IEEE Trans. Pattern Anal. Mach. Intell.* **43**(2), 652–662. 10.1109/TPAMI.2019.2938758 (2021). 31484108 10.1109/TPAMI.2019.2938758

[38] Wang, Z., Wu, Y., Wang, J., Xu, J. & Shao, W. Res2Fusion: Infrared and visible image fusion based on dense Res2net and double nonlocal attention models. *IEEE Trans. Instrum. Meas.* **71**, 1–12. 10.1109/TIM.2021.3139654 (2022).

[39] Tang, L., Xiang, X., Zhang, H., Gong, M. & Ma, J. DIVFusion: Darkness-free infrared and visible image fusion. *Inf. Fusion* **91**, 477–493. 10.1016/j.inffus.2022.10.034 (2023).

[40] Li, J., Jiang, J., Liang, P., Ma, J. & Nie, L. MaeFuse: Transferring omni features with pretrained masked autoencoders for infrared and visible image fusion via guided training. *IEEE Trans. Image Process.* **34**, 1340–1353. 10.1109/TIP.2025.3541562 (2025). 40031430 10.1109/TIP.2025.3541562

[41] Zhang, Z. et al. GuideFuse: A novel guided auto-encoder fusion network for infrared and visible images. *IEEE Trans. Instrum. Meas.* **73**, 1–11. 10.1109/TIM.2023.3306537 (2024).

[42] Gu, A. et al. Combining recurrent, convolutional, and continuous-time models with linear state space layers. *Adv. Neural Inf. Process. Syst.* **34**, 572–585. 10.48550/arXiv.2110.13985 (2021).

[43] Gu, A., Goel, K., & Ré, C. Efficiently Modeling Long Sequences with Structured State Spaces. In* Proceedings of the International Conference on Learning Representations (ICLR)*. (2022) 10.48550/arXiv.2111.00396

[44] Zhang, T., et al. CWNet: Causal wavelet network for low-light image enhancement. *arXiv preprint,* (2025). 10.48550/arXiv.2507.10689

[45] Dong, W. et al. Fusion-Mamba for cross-modality object detection. *IEEE Trans. Multimed.* **27**, 7392–7406. 10.1109/TMM.2025.3599020 (2025).

[46] Zhou, J. et al. MaDiNet: Mamba diffusion network for SAR target detection. *IEEE Trans. Circuits Syst. Video Technol.* **35**, 10787–10800. 10.1109/TCSVT.2025.3574657 (2025).

[47] Li, Z., Pan, H., Zhang, K., Wang, Y., & Yu, F. MambaDFuse: A Mamba-based dual-phase model for multi-modality image fusion. Preprint at. 10.1016/j.inffus.2025.103414. (2024).

[48] Xu, G. et al. Haar wavelet downsampling: A simple but effective downsampling module for semantic segmentation. *Pattern Recogn.* **143**, 109819. 10.1016/j.patcog.2023.109819 (2023).

[49] Yang, Y., Yuan, G. & Li, J. SFFnet: A wavelet-based spatial and frequency domain fusion network for remote sensing segmentation. *IEEE Trans. Geosci. Remote Sens.* **62**, 1–15. 10.1109/TGRS.2024.3427370 (2024).

[50] Wang, Z. et al. FreqGAN: Infrared and visible image fusion via unified frequency adversarial learning. *IEEE Trans. Circuits Syst. Video Technol.* **35**, 728–740. 10.1109/TCSVT.2024.3460172 (2025).

[51] Finder, S. E., Amoyal, R., Treister, E., & Freifeld, O. Wavelet convolutions for large receptive fields. In *European Conference on Computer Vision*, 2025, 363–380. 10.1007/978-3-031-72949-2_21

[52] Sang, H. & Peng, X. Nighttime pedestrian detection using crossed wavelet fusion networks. *J. Electron. Imaging* **34**(2), 023067. 10.1117/1.JEI.34.2.023067 (2025).

[53] Zhu, H., Dong, W., Yang, L., et al. WaveMamba: Wavelet-Driven Mamba Fusion for RGB-Infrared Object Detection, Preprint at 10.48550/arXiv.2507.18173. (2025).

[54] Chen, X. et al. Mask-guided frequency feature fusion for visible–infrared remote sensing object detection. *IEEE Trans. Geosci. Remote Sens.* **63**, 1–15. 10.1109/TGRS.2025.3612495 (2025).

[55] Liu, Y. X., Li, W. J., Liu, L., et al. ATRNet-STAR: A large dataset and benchmark towards remote sensing object recognition in the wild. *arXiv preprint*, https://arxiv.org/abs/2501.13354. (2025). 10.1109/TPAMI.2026.3658649 41605166

[56] Diao, W. et al. Dual-conditionally guided diffusion models for fusion of unregistered multisource remote sensing images. *IEEE Trans. Geosci. Remote Sens.* **63**(000), 1–17. 10.1109/TGRS.2025.3577073 (2025).

[57] Jie, Y. et al. Fs-diff: Semantic guidance and clarity-aware simultaneous multimodal image fusion and super-resolution. *Inf. Fusion* **121**, 103146. 10.1016/j.inffus.2025.103146 (2025).

[58] Yang, B. et al. Lfdt-fusion: A latent feature-guided diffusion transformer model for general image fusion. *Inf. Fusion*10.1016/j.inffus.2024.102639 (2025).

[59] Zhu, Q. et al. GDSR: Global-detail integration through dual-branch network with wavelet losses for remote sensing image super-resolution. *IEEE Trans. Geosci. Remote Sens.* **63**, 1–16. 10.1109/TGRS.2025.3617006 (2025).

[60] Li, J. Y., Zhang, Z. L., & Zuo, W. M. Rethinking transformer-based blind-spot network for self-supervised image denoising. Preprint at 10.48550/arXiv.2404.07846. (2025).

[61] Guo, J., Du, H., Hao, X. & Zhang, M. CFET: A cross-fusion enhanced transformer for visible-infrared person re-identification. *Expert Syst. Appl.* **271**, 126645. 10.1016/j.eswa.2025.126645 (2025).

[62] Liu, Z., Lin, Y., Cao, Y., et al. Swin Transformer: Hierarchical vision transformer using shifted windows. In* IEEE/CVF International Conference on Computer Vision (ICCV)*, (2021). 10.48550/arXiv.2103.14030

[63] Wang, Z., Bovik, A. C., Sheikh, H. R. & Simoncelli, E. P. Image quality assessment: From error visibility to structural similarity. *IEEE Trans. Image Process.* **13**(4), 600–612. 10.1109/TIP.2003.819861 (2004). 15376593 10.1109/tip.2003.819861

[64] Lin, T.Y., Maire, M., Belongie, S., et al. Microsoft COCO: Common objects in context. *In Computer Vision – ECCV 2014*. 740–755. (2014). 10.1007/978-3-319-10602-1_48

[65] Jia, X., Zhu, C., Li, M., Tang, W., & Zhou, W. LLVIP: A visible-infrared paired dataset for low-light vision. In *2021 IEEE/CVF International Conference on Computer Vision Workshops (ICCVW)*, 2021, 3489–3497. 10.1109/ICCVW54120.2021.00389

